# Identification rate of *Legionella* species in non-purulent sputum culture is comparable to that in purulent sputum culture in *Legionella* pneumonia

**DOI:** 10.1128/jcm.01665-23

**Published:** 2024-03-19

**Authors:** Akihiro Ito, Tadashi Ishida, Hiromasa Tachibana, Yosuke Nakanishi, Masanori Kawataki, Akio Yamazaki, Yasuyoshi Washio

**Affiliations:** 1 Department of Respiratory Medicine, Ohara Healthcare Foundation, Kurashiki Central Hospital, Kurashiki, Japan; 2 Department of Respiratory Medicine, National Hospital Organization, Minami Kyoto Hospital, Kyoto, Japan; 3 Department of Respiratory Medicine, Shiga University of Medical Science, Otsu, Japan; 4 Research Institute for Diseases of the Chest, Graduate School of Medical Sciences, Kyushu University, Fukuoka, Japan; Medical College of Wisconsin, Milwaukee, Wisconsin, USA

**Keywords:** diagnosis, *Legionella *pneumonia, predictive factor, purulent sputum, sputum culture

## Abstract

Many *Legionella* pneumonia patients do not produce sputum, and it is unknown whether purulent sputum is required for the identification of *Legionella* species. This study aimed to evaluate the identification rate of *Legionella* species based on sputum quality and the factors predictive of *Legionella* infection. This study included *Legionella* pneumonia patients at Kurashiki Central Hospital from November 2000 to December 2022. Sputum quality, based on gram staining, was classified as the following: Geckler 1/2, 3/6 and 4/5. Geckler 4/5 was defined as purulent sputum. The sputa of 104 of 124 *Legionella* pneumonia patients were cultured. Fifty-four patients (51.9%) were identified with *Legionella* species, most of which were *Legionella pneumophila* serogroup 1 (81.5%). The identification rates of *Legionella* species according to sputum quality were 57.1% (16/28) in Geckler 1/2 sputum, 50.0% (34/68) in Geckler 3/6 sputum, and 50.0% (4/8) in Geckler 4/5 sputum, which were not significantly different (*P* = 0.86). On multivariate analysis, pre-culture treatment with anti-*Legionella* antimicrobials (odds ratio [OR] 0.26, 95% confidence interval [CI] 0.06–0.91), Pneumonia Severity Index class ≥IV (OR 2.57 [95% CI 1.02–6.71]), and intensive care unit admission (OR 3.08, 95% CI 1.06–10.09) correlated with the ability to identify *Legionella* species, but sputum quality did not (OR 0.88, 95% CI 0.17–4.41). The identification rate of *Legionella* species in non-purulent sputum was similar to that in purulent sputum. For the diagnosis of *Legionella* pneumonia, sputum should be collected before administering anti-*Legionella* antibiotics and cultured regardless of sputum quality.

## INTRODUCTION

*Legionella* pneumonia reportedly accounts for 1% to 10% of cases of community-acquired pneumonia (CAP) (1–5). In fact, the percentage increases by 10% to 15% in severe CAP, and *Legionella* species are one of the important causative pathogens of CAP following *Streptococcus pneumoniae* (6–8). Delay of administration of anti-*Legionella* antimicrobials for *Legionella* pneumonia is reported to be a risk factor for poor prognosis (9), suggesting that early diagnosis of *Legionella* pneumonia and its treatment by appropriate antibiotics is essential.

Globally, the urinary antigen test (UAT) is widely used for diagnosing *Legionella* pneumonia (10–13), due to the simplicity of the procedure and rapid results. However, since the *Legionella* UAT has high specificity (99%) but only moderate sensitivity (74%) (14), the possibility of *Legionella* pneumonia infection should not be denied based on negative UAT results. The main reason for false-negative results with the existing *Legionella* UAT is the inability to identify *Legionella* species other than *L. pneumophila* serogroup (SG) 1. Therefore, culture of lower respiratory tract specimens, including sputum, is needed for detecting all SGs of *L. pneumophila,* including SG1 and other *Legionella* species in cases with both positive and negative UAT results.

Although culture of *Legionella* species from a respiratory specimen is the diagnostic gold standard (15), it is known to have certain drawbacks, including the fact that it needs a specific culture medium, such as buffered charcoal yeast extract-α (BCYE-α) and Wadowsky-Yee-Okuda-α (WYO-α), and takes about 3–5 days to identify the *Legionella* species (10). In addition, previous reports showed that 50% to 70% of *Legionella* pneumonia patients do not produce sputum (16–18). We have also often experienced the inability to collect purulent sputum from *Legionella* pneumonia patients. For identification of the causative microorganisms in sputum culture, appropriate collection and culture of purulent sputum is essential, as far as possible. However, since there is much variability in the production of sputum as a symptom and the quality of sputum, whether purulent, in *Legionella* pneumonia cases, the detection power of respective sputum purulence in *Legionella* pneumonia is unknown.

The aim of the present study was to evaluate the occurrence of sputum purulence and detection ability of *Legionella* species based on sputum quality, in addition to factors predictive of the identification of *Legionella* species in *Legionella* pneumonia patients.

## MATERIALS AND METHODS

### Study population

*Legionella* pneumonia patients diagnosed at Kurashiki Central Hospital, a 1,166-bed tertiary hospital, from November 2000 to December 2022 were retrospectively enrolled in this study. Patients who had abnormal shadows on radiologic examinations, along with at least one symptom, such as fever, cough, sputum, chest pain, and general malaise, and one clinical finding (abnormal auscultation findings or an increased inflammatory reaction) were diagnosed with pneumonia (19). Patients who were <15 years old and those with hospital-acquired pneumonia were excluded. This study was approved by the institutional review board of our hospital (IRB number 4168). The IRB waived the need for obtaining patient informed consent due to the retrospective nature of the study.

### Study design

We investigated the clinical characteristics of *Legionella* pneumonia patients, including age, sex, comorbidities, symptoms, pre-culture antibiotic treatment before admission, and severity of pneumonia, including CURB-65 score [confusion, urea >7 mmol/L, respiratory rate ≥30 breaths per minute, low blood pressure (systolic <90 mmHg or diastolic ≤60 mmHg), and age ≥65 years] (20), Pneumonia Severity Index (PSI) [calculated using the total score of age, sex, resident in nursing home or not, five comorbidities, five physical examination findings, six laboratory findings, and one radiological finding] (21), A-DROP score [age ≥70 y in men and ≥75 y in women, dehydration or blood urea nitrogen ≥21 mg/dl, SpO_2_ ≤90%, disturbance in orientation, and systolic blood pressure ≤90 mmHg] (22), and prognosis, in addition to sputum quality and culture results. *Legionella* pneumonia was diagnosed in patients who satisfied at least one of the following criteria: positive results of *Legionella* UATs, identification of *Legionella* species in lower respiratory tract sputum samples, positive results of *Legionella* gene tests, or 4-fold increase in paired serum antibody testing. In clinical practice at our hospital, Binax NOW *Legionella* (Abbott Diagnostics Medical, Lake Forest, CA, USA) (Binax) was used from December 2004 to July 2016, and Immunocatch *Legionella* (Eiken Kagaku Corporation, Tokyo, Japan) has been used since July 2016 for the diagnosis of *Legionella* infection. Since June 2019, Libotest *Legionella* (Kyokutou Corporation, Tokyo, Japan) (Libotest) has also been used in addition to Immunocatch *Legionella*.

In daily clinical practice, sputum culture using WYO-α, gene testing by loop-mediated isothermal amplification (LAMP) (Eiken Kagaku Corporation, Tokyo, Japan) of sputum, and serum antibody tests were performed at the discretion of the attending physicians.

### Evaluation of sputum quality and sputum culture method

Sputum quality was evaluated by experienced microbiological test engineers using Geckler’s classification (23), in which gram-stained sputum smears are rated as Geckler classes 1–6, based on the number of buccal squamous epithelial cells and leukocytes (Geckler 1, buccal squamous epithelial cells > 25 and leukocytes < 10; Geckler 2, buccal squamous epithelial cells > 25 and leukocytes 10–25; Geckler 3, buccal squamous epithelial cells > 25 and leukocytes > 25; Geckler 4, buccal squamous epithelial cells 10–25 and leukocytes > 25; Geckler 5, buccal squamous epithelial cells < 10 and leukocytes > 25; and Geckler 6, buccal squamous epithelial cells < 25 and leukocytes < 25). In the present study, we defined Geckler 4/5 as purulent sputum and the classes other than Geckler 4/5 as non-purulent sputum, as previously described (24). For sputum culture, we used WYO-α medium and acid pre-treatment of sputum to decrease bacterial contamination of the sputum from patients suspected to have *Legionella* pneumonia.

### Study outcomes

The primary outcome was the identification rate of *Legionella* species in each sputum quality class according to Geckler’s classification. The secondary outcome was the identification rate of *Legionella* species in terms of pneumonia severity, based on CURB-65, PSI, and A-DROP scores. CURB-65 ≥3 points, PSI ≥ class IV, and A-DROP ≥3 points were defined as severe pneumonia (20–22). In addition, we evaluated factors predictive of the identification of *Legionella* species on sputum culture.

### Statistical analysis

Continuous variables are expressed as medians and interquartile ranges, and categorical variables are expressed as numbers and percentages. Categorical variables were analyzed by Fisher’s exact test, and continuous variables by the non-parametric Mann-Whitney U-test. To evaluate the identification ability according to sputum quality, we divided sputum into the following three quality groups: Geckler 1/2, 3/6, and 4/5. Univariate analysis was performed to identify predictive factors in the identification of *Legionella* species on sputum culture. Multivariate analysis using stepwise logistic regression analysis was conducted for all variables that were found to have a *P* value of ≤ 0.05 on univariate analysis, in addition to sputum quality and pre-culture treatment using anti-*Legionella* antibiotics. All tests were two-tailed, and a *P* value of < 0.05 was considered significant. All statistical analyses were performed using EZR statistical software (version 3.0.3, Vienna, Austria) (25).

## RESULTS

### Patients’ baseline characteristics

**Fig 1:**
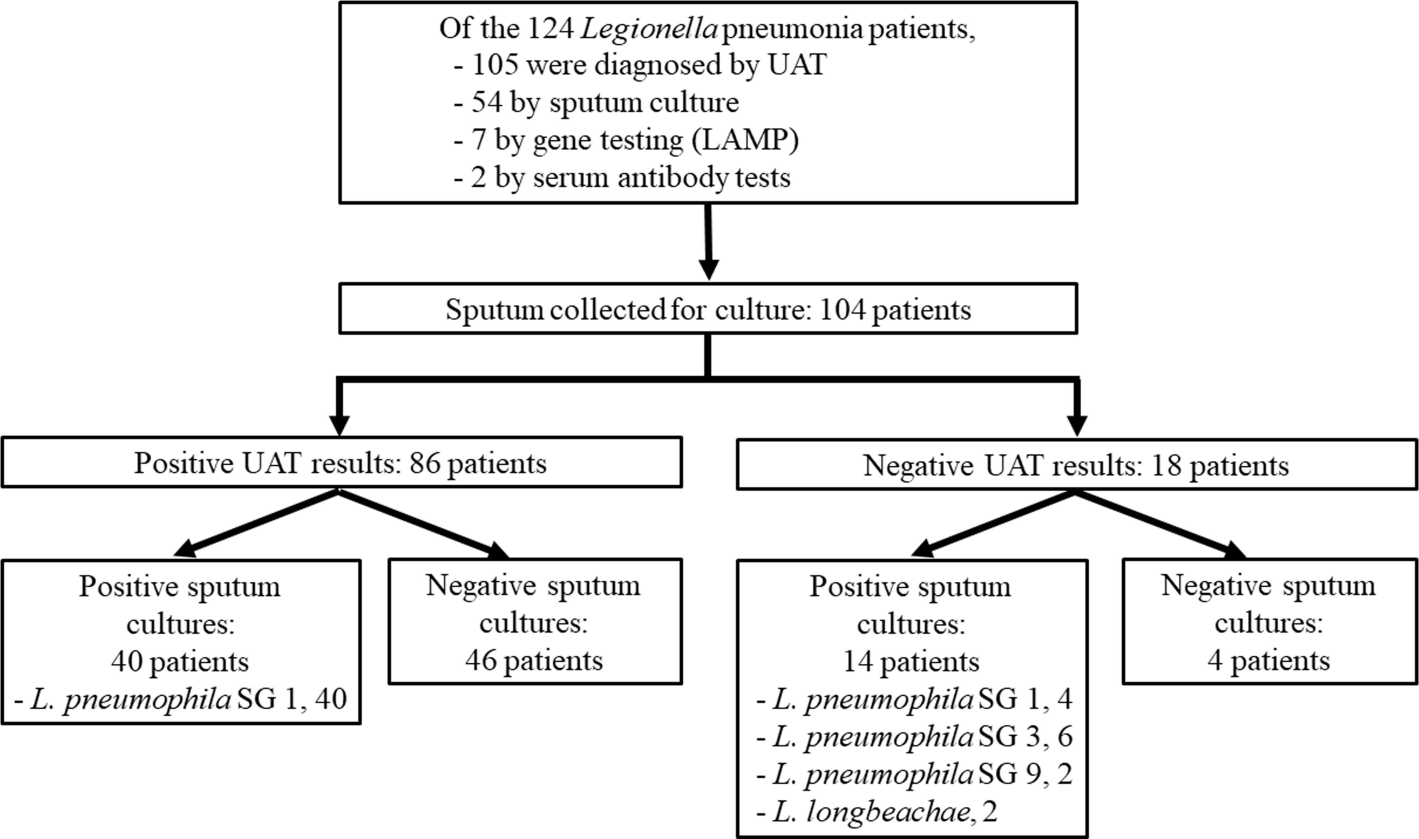
Study flowchart, the number of positive results, and the results of diagnostic tests, including urinary antigen tests, sputum cultures, gene tests, and serum antibodies, among the 124 *Legionella* pneumonia patients are shown. LAMP, loop-mediated isothermal amplification; SG, serogroup; UAT, urinary antigen test.

A total of 124 *Legionella* pneumonia patients, including 119 hospitalized patients and 5 outpatients, were evaluated. Figure 1 shows the results of *Legionella* UAT, culture, sputum gene analyses by LAMP, and serum antibodies. Among the 124 *Legionella* pneumonia patients, sputum for culture was obtained from 104 patients (83.9%). The sputum culture positivity rate was 51.9% (54/104), and 14 among the 18 patients with negative UAT results were diagnosed by sputum culture (Fig. 1). The baseline clinical characteristics of *Legionella* pneumonia patients with and without the identification of *Legionella* species by sputum culture are shown in Table 1. Median patient age was 68 years, and 87.5% were male. The most common comorbidity was diabetes mellitus, followed by chronic heart disease. The most common symptom was fever (89.4%), followed by cough (39.4%) and relative bradycardia (39.4%). Forty-six patients (53.5%) without sputum as a symptom were identified with *Legionella* by sputum culture. LAMP was performed in 10 of the 104 patients, among whom 7 patients (70%) showed positive results. Regarding the positivity rate following LAMP in terms of sputum quality, the LAMP positive rate was 100% (1/1) in purulent sputum and 66.7% (6/9) in non-purulent sputum.

**TABLE 1:** Baseline clinical characteristics of *Legionella* pneumonia patients with positive and negative sputum culture results^*b*^^,*c*^

	All patients *n* = 104	Positive sputum culture *n* = 54	Negative sputum culture *n* = 50	*P* value
Age (y)	68 [60–76]	66 [58–73]	70 [65–78]	0.043
Male	91 (87.5)	51 (94.4)	40 (80.0)	0.037
Smoking status				
Current +past	83 (79.8)	43 (79.6)	40 (80.0)	0.706
Comorbidities				
Chronic obstructive pulmonary disease	8 (7.7)	3 (5.6)	5 (10.0)	0.477
Diabetes mellitus	30 (28.8)	18 (33.3)	12 (24.0)	0.387
Chronic heart disease	19 (18.3)	12 (22.2)	7 (14.0)	0.318
Malignancy	7 (6.7)	4 (7.4)	3 (6.0)	1.000
Chronic renal disease	8 (7.7)	6 (11.1)	2 (4.0)	0.273
Chronic liver disease	8 (7.7)	5 (9.3)	3 (6.0)	0.717
Cerebrovascular disease	14 (13.5)	5 (9.3)	9 (18.0)	0.254
Symptoms				
Fever	93 (89.4)	46 (85.2)	47 (94.0)	0.096
Cough	41 (39.4)	18 (33.3)	23 (46.0)	0.226
Sputum	18 (17.3)	8 (14.8)	10 (20.0)	0.604
Dyspnea	24 (23.1)	17 (31.5)	7 (14.0)	0.061
Headache	15 (14.4)	7 (13.0)	8 (16.0)	0.781
Abdominal pain	0 (0)	0 (0)	0 (0)	NA
Diarrhea	10 (9.6)	5 (9.3)	5 (10.0)	1.000
Arthralgia	4 (3.8)	3 (5.6)	1 (2.0)	0.619
Myalgia	2 (1.9)	1 (1.9)	1 (2.0)	1.000
Mental disturbance	34 (32.7)	17 (31.5)	17 (34.0)	0.834
Vital signs				
Temperature (°C)	38.9 [38.0–39.5]	39.0 [38.0–39.5]	38.7 [38.0–39.4]	0.571
Heart rate (beats/min)	100 [90–114]	105 [86–121]	99 [91–110]	0.307
Relative bradycardia	41 (39.4)	19 (35.2)	22 (44.0)	0.424
Systolic blood pressure (mmHg)	136 [120–156]	137 [120–156]	133 [119–152]	0.925
Laboratory findings				
C-reactive protein (mg/L)	250.6 [185.7–320.0]	269.4 [201.3–337.3]	230.0 [152.9–288.4]	0.048
Albumin (g/dL)	2.9 [2.4–3.3]	2.9 [2.4–3.2]	2.9 [2.5–3.3]	0.643
Aspartate aminotransferase (U/L)	52 [29–158]	57 [30–181]	51 [28–150]	0.683
Alanine aminotransferase (U/L)	40 [21–67]	41 [22–63]	39 [20–71]	0.943
Lactate dehydrogenase (U/L)	317 [227–489]	307 [226–539]	335 [240–443]	0.940
Blood urea nitrogen (mg/dL)	21 [15–29]	21 [14–34]	21 [15–26]	0.592
Creatinine (mg/dL)	1.02 [0.82–1.52]	1.15 [0.86–2.04]	0.98 [0.78–1.27]	0.031
Sodium (mmol/L)	133 [130–138]	133 [129–136]	134 [131–139]	0.271
White blood cells (×10^3^ /µL)	11.0 [8.6–14.4]	11.3 [9.5–14.7]	10.5 [7.8–13.8]	0.332
Platelets (×10^4^ /µL)	17.2 [12.7–21.8]	15.8 [12.0–20.0]	18.5 [13.1–24.5]	0.080
Pre-treatment with anti-*Legionella* antibiotics	15 (14.4)	5 (9.3)	10 (20.0)	0.164
Severity of pneumonia				
CURB-65 (points)				0.961
0	6 (5.8)	4 (7.4)	2 (4.0)	
1	35 (33.7)	18 (33.0)	17 (34.0)	
2	32 (30.8)	17 (31.5)	15 (30.0)	
3	23 (22.1)	11 (20.4)	12 (24.0)	
4	8 (7.7)	4 (7.4)	4 (8.0)	
5	0 (0)	0 (0)	0 (0)	
CURB-65 ≥ 3 points	31 (29.8)	15 (27.8)	16 (32.0)	0.673
PSI (points)	99 [86–125]	105 [96–124]	95 [78–128]	0.176
PSI (class)				0.006
I	2 (1.9)	1 (1.9)	1 (2.0)	
II	10 (9.6)	1 (1.9)	9 (18.0)	
III	21 (20.2)	9 (16.7)	12 (24.0)	
IV	49 (47.1)	33 (61.1)	16 (32.0)	
V	22 (21.2)	10 (18.5)	12 (24.0)	
PSI class ≥IV	71 (68.3)	43 (79.6)	28 (56.0)	0.012
A-DROP (points)				0.509
0	12 (11.5)	4 (7.4)	8 (16.0)	
1	35 (33.7)	20 (37.0)	15 (30.0)	
2	25 (24.0)	15 (27.8)	10 (20.0)	
3	23 (22.1)	11 (20.4)	12 (24.0)	
4	8 (7.7)	3 (5.6)	5 (10.0)	
5	1 (1.0)	1 (1.9)	0 (0)	
A-DROP ≥3 points	32 (30.8)	15 (27.8)	17 (34.0)	0.529
ICU admission	38 (36.5)	28 (51.9)	10 (20.0)	0.001
ICU admission within 24 hours of admission	26 (25.0)	19 (35.2)	7 (14.0)	0.014
Late ICU admission^*a*^	12 (11.5)	9 (16.7)	3 (6.0)	0.126
30-day mortality	6 (5.8)	3 (5.6)	3 (6.0)	1.000
In-hospital mortality	7 (6.7)	3 (5.6)	4 (8.0)	0.708

^*a*^ Late ICU admission means ICU admission more than 24 h after admission.

^*b*^ A-DROP: age ≥70 years in men or ≥75 years in women, blood urea nitrogen ≥21 mg/dL or dehydration, oxyhemoglobin saturation measured by pulse oximetry ≤90% or partial pressure of oxygen in arterial blood ≤60 mmHg, confusion, and systolic blood pressure ≤90 mmHg; CURB-65: confusion, urea >7 mmol/L, respiratory rate ≥30 breaths/min, low blood pressure (systolic <90 mmHg or diastolic ≤60 mmHg), and age ≥65 y; ICU: intensive care unit; NA: not assessed; PSI: Pneumonia Severity Index.

^*c*^ Data are shown as numbers (%) or medians and interquartile range.

### Distribution of *Legionella* species and *Legionella pneumophila* serogroups identified by sputum culture

**Fig 2:**
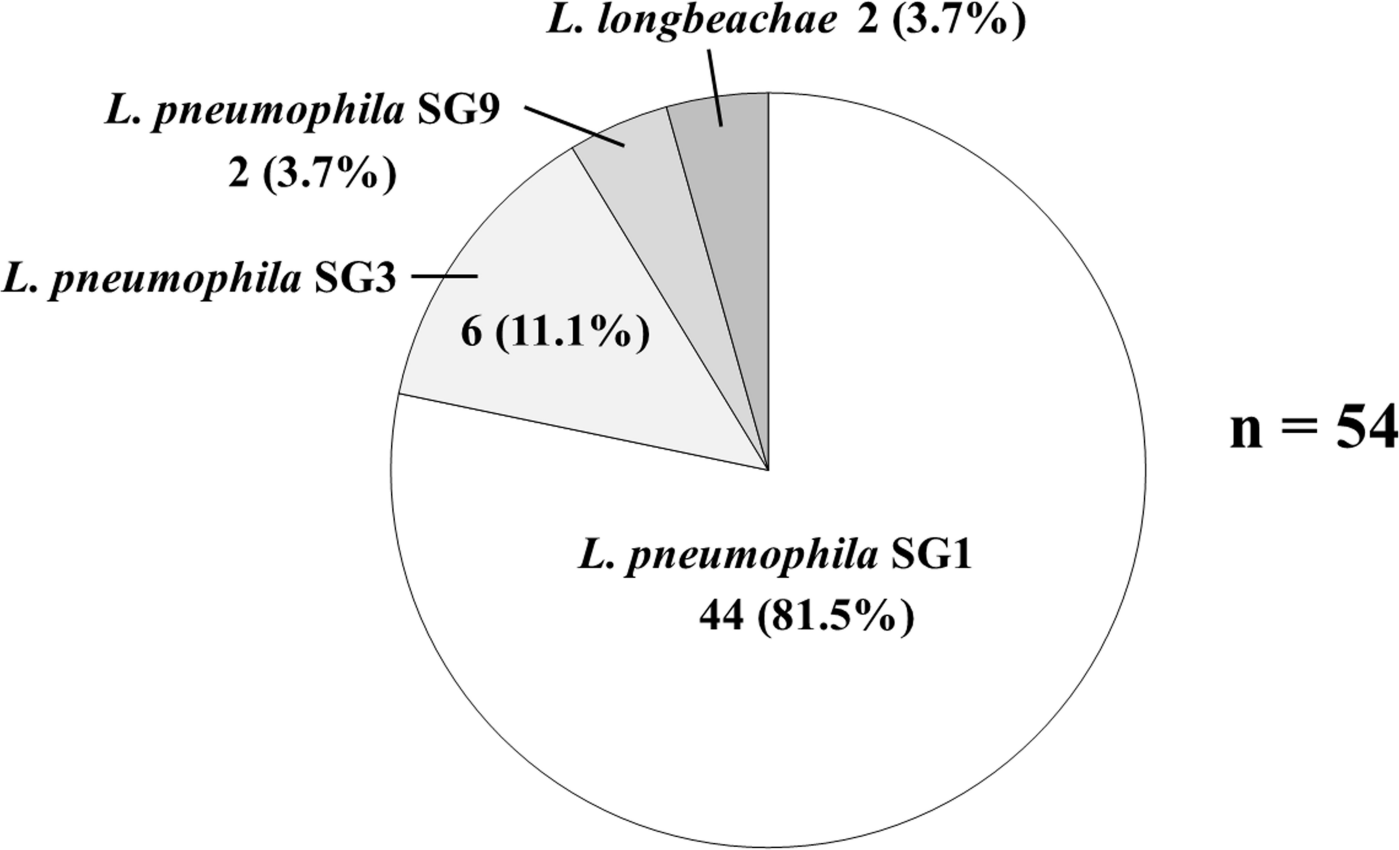
Distribution of *Legionella* species and *Legionella pneumophila* serogroups in sputum culture.

The *Legionella* species identified by sputum culture is shown in Fig. 2. The most common *Legionella* species was *L. pneumophila* SG1 (81.5%), followed by *L. pneumophila* SG3 (11.1%). Non-*L*. *pneumophila* SG1 was identified in 18.5% of *Legionella* pneumonia patients.

### Ability to identify *Legionella* species by sputum culture in terms of sputum quality

**Fig 3:**
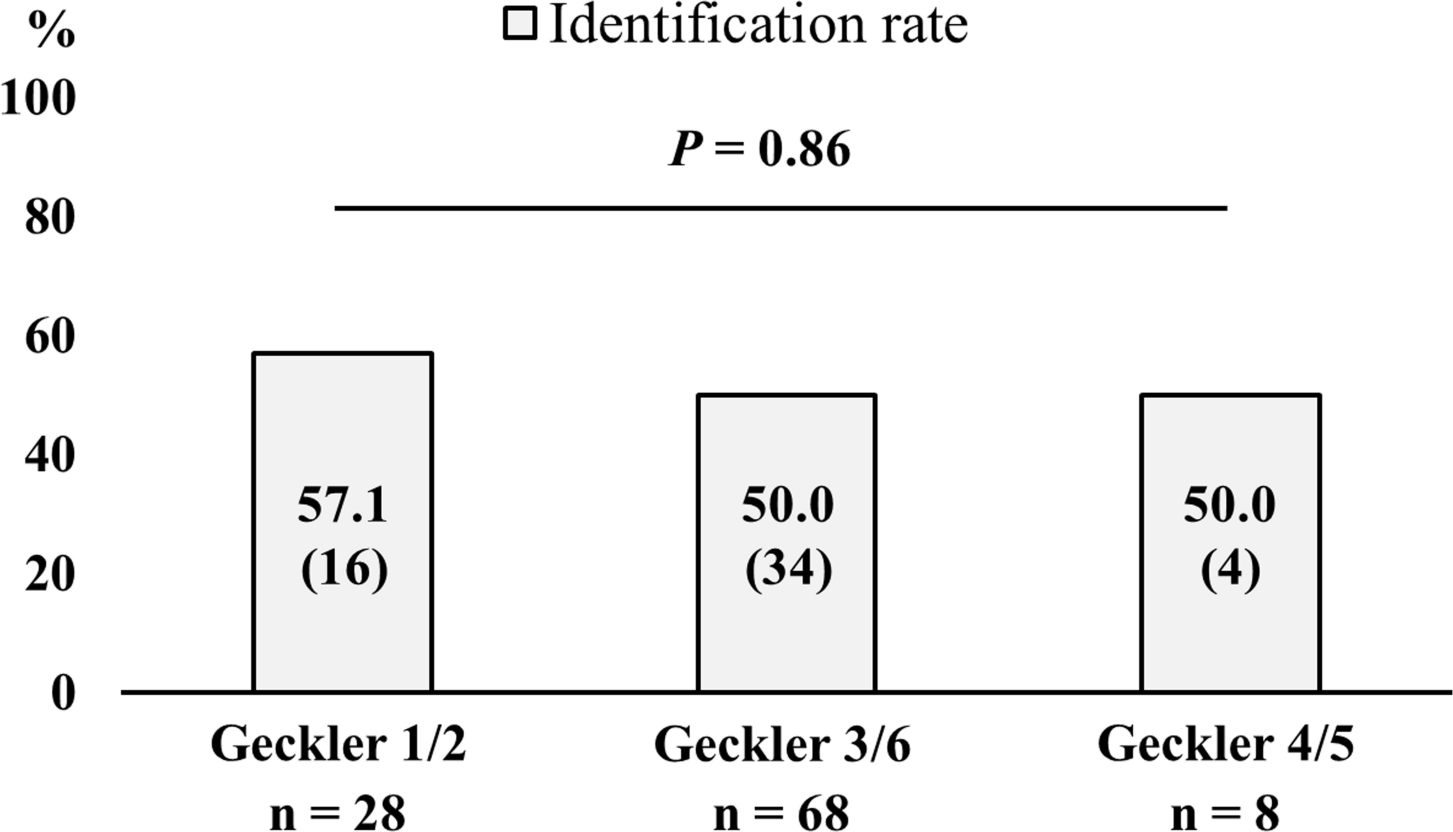
Identification rate of *Legionella* species according to sputum quality in the 104 *Legionella* pneumonia patients. Almost all the patients (92.3%) had non-purulent sputum, and a few patients (7.7%) had purulent sputum. The identification rate of *Legionella* species was not significantly different in each sputum quality group (*P* = 0.86).

Figure 3 shows the identification rate of *Legionella* infection in the respective sputum quality groups. Although a few patients had purulent sputum (8/104, 7.7%), most patients did not (96/104, 92.3%). *Legionella* species were most identified in Geckler 3/6 sputum (34/54, 63.0%), followed by Geckler 1/2 (16/54, 29.6%) and 4/5 (4/54, 7.4%) sputa. Regarding the identification rate of *Legionella* species, there were no significant differences in terms of sputum quality (*P* = 0.86). In patients with purulent sputum, *Streptococcus pneumoniae* was cultivated in only one patient (1/8), whereas no significant pathogens were cultivated in the others, including in four patients identified with *Legionella* species.

### Ability to identify *Legionella* species by sputum culture according to pneumonia severity

Table 2 shows the identification rate of *Legionella* species in non-severe and severe pneumonia groups according to CURB-65, PSI, and A-DROP scores. There was a significant difference in the ability to identify *Legionella* according to the severity of pneumonia determined using PSI, but not with CURB-65 and A-DROP scores.

**TABLE 2:** Identification rate of *Legionella* species by sputum culture in terms of pneumonia severity^*a*^^,*b*^

	All patients *n* = 104	Patients identified with *Legionella* infection *n* = 54	Identification rate of *Legionella* species in each pneumonia severity group %	*P* value
CURB-65 (points)				0.673
0–2	73 (70.2)	39 (72.2)	53.4	
3–5	31 (29.8)	15 (27.8)	48.4	
PSI (class)				0.012
I-III	33 (31.7)	11 (20.4)	33.3	
IV-V	71 (68.3)	43 (79.6)	60.6	
A-DROP (points)				0.529
0–2	72 (69.2)	39 (72.2)	54.2	
3–5	32 (30.8)	15 (27.8)	46.9	

^*a*^ Data are shown as numbers (%).

^*b*^ A-DROP: age ≥70 years in men or ≥75 years in women, blood urea nitrogen ≥21 mg/dL or dehydration, oxyhemoglobin saturation measured by pulse oximetry ≤90% or partial pressure of oxygen in arterial blood ≤60 mmHg, confusion, and systolic blood pressure ≤90 mmHg; CURB-65: confusion, urea >7 mmol/L, respiratory rate ≥30 breaths/min, low blood pressure (systolic <90 mmHg or diastolic ≤60 mmHg), and age ≥65 y; PSI: Pneumonia Severity Index.

### Predictive factors for identifying *Legionella* species by sputum culture

Sputum purulence was not a significant predictive factor in both univariate and multivariate analyses. In multivariate analysis, pre-culture treatment with anti-*Legionella* antibiotics was a negative predictive factor (odds ratio [OR] 0.26 [95% confidence interval (CI) 0.06–0.91], *P* = 0.044), whereas PSI class ≥IV (OR 2.57 [95% CI 1.02–6.71], *P* = 0.048) and intensive care unit (ICU) admission within 24 h after admission (OR 3.08 [95% CI 1.06–10.09], *P* = 0.048) were both positive predictive factors for the identification of *Legionella* species (Table 3).

**TABLE 3:** Predictive factors in the identification of *Legionella* species by sputum culture^*a*^

	Univariate analysis		Multivariate analysis	
	Odds ratio (95% CI)	*P* value	Odds ratio (95% CI)	*P* value
Age	0.97 [0.94–1.00]	0.099		
Sex	4.25 [1.21–19.90]	0.036		
PSI class ≥IV	3.07 [1.31–7.52]	0.011	2.57 [1.02–6.71]	0.048
ICU admission within 24 h	3.33 [1.30–9.38]	0.015	3.08 [1.06–10.09]	0.048
Pre-treatment with anti-*Legionella* antibiotics	0.41 [0.12–1.25]	0.127	0.26 [0.06–0.91]	0.044
Sputum quality	1.22 [0.61–2.48]	0.569	0.88 [0.17–4.41]	0.877

^*a*^ CI, confidence interval; ICU, intensive care unit; NA, not assessed; PSI, Pneumonia Severity Index.

**Fig 4:**
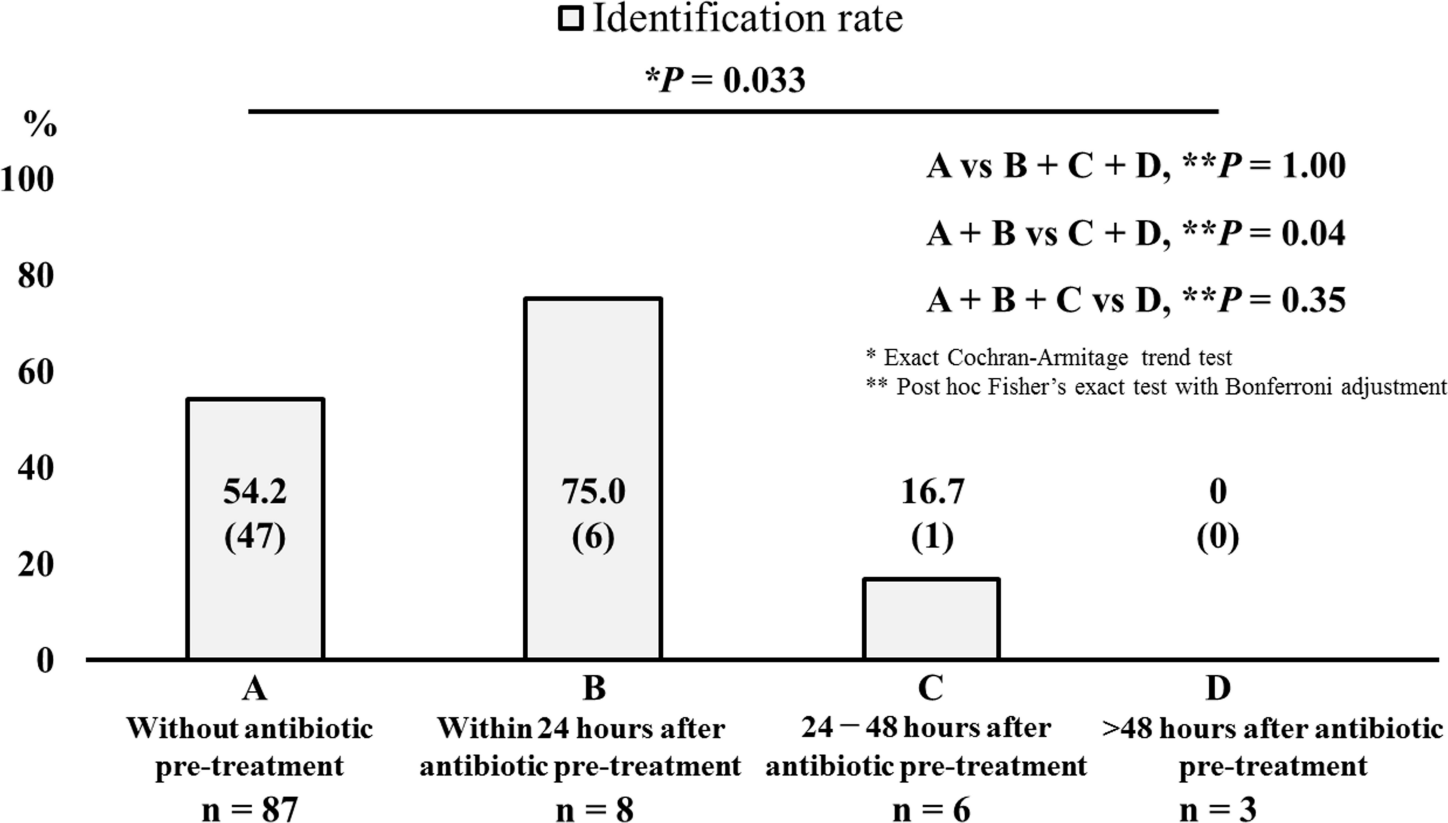
Identification rate of *Legionella* species by sputum culture relative to time after the administration of anti-*Legionella* antibiotics The identification rate of *Legionella* species was 54.2% (47/87) in patients without pre-culture antibiotic treatment using anti-*Legionella* antibiotics, 75.0% (6/8) in patients within 24 h after the administration of anti-*Legionella* antibiotics, 16.7% (1/6) in patients within 24 to 48 h after the administration of anti-*Legionella* antibiotics, and 0% (0/3) in patients more than 48 h after the administration of anti-*Legionella* antibiotics.

Regarding the correlation between the identification rate of *Legionella* species and interval between sputum collection and administration of anti-*Legionella* antimicrobials, the identification rate was not inferior in patients in whom sputum was sampled within 24 h after administering anti-*Legionella* antibiotics compared with patients who did not receive antibiotic therapy for *Legionella* pneumonia [75.0% (6/8) vs 54.2% (47/87)]. However, the identification rate of *Legionella* species decreased steeply and was significantly lower if the sputum was collected more than 24 h after administering anti-*Legionella* antibiotics, compared with patients without pre-treatment and with pre-treatment by anti-*Legionella* antibiotics within 24 h (55.8% vs 11.1%, *p* = 0.04) (Fig. 4).

## DISCUSSION

The present study showed that most *Legionella* pneumonia patients had non-purulent sputum, and the identification rate of *Legionella* species by sputum culture did not differ significantly between purulent and non-purulent sputa. Regarding the correlation between the identification rate of *Legionella* species and pneumonia severity, the identification rate was higher in severe pneumonia patients, defined by PSI, than in non-severe patients. In addition, antibiotic treatment for *Legionella* pneumonia before sputum sampling was a negative predictor, and severe pneumonia defined by PSI and ICU admission within 24 h after admission were positive predictors in the identification of *Legionella* species by sputum culture.

Many *Legionella* pneumonia patients have non-purulent sputum or sputum cannot be obtained for culture (4). Therefore, most attending physicians usually use UAT kits for the diagnosis of Legionellosis in Japan and many other countries (10–13). The main disadvantage of the existing UAT kits is their inability to diagnose *Legionella* pneumonia due to non-*Legionella pneumophila* serogroup 1 (26). In February 2019, Libotest *Legionella* (Kyokutou Corporation, Tokyo, Japan), which can detect all *Legionella pneumophila* serogroups, was launched in Japan. We previously reported that Libotest *Legionella* had similar diagnostic ability compared with existing UAT kits, including BinaxNOW and Q line, in addition to the ability to detect all serogroups of *Legionella pneumophila* (18). However, since the sensitivity of Libotest *Legionella* for diagnosing Legionellosis due to non-*L*. *pneumophila* SG1 is unknown and there is a substantial need for surveillance of *Legionella* species in *Legionella* pneumonia patients, performance of sputum culture for the diagnosis of *Legionella* pneumonia will continue to be important. In the present study, sputum culture was performed in 83.9% of *Legionella* pneumonia patients, and *Legionella* species were identified in almost half of them, although only 17.5% of the patients had sputum as a symptom. In addition, the identification rate of *Legionella* species was not significantly different between purulent and non-purulent sputa (50.0% vs 52.1%, *p* = 1.00). Cunha et al. reported that the sputum of *Legionella* pneumonia patients had less neutrophils and was watery (10). According to these studies, including that of our study, efforts should be made to collect sputum for the diagnosis of *Legionella* pneumonia even in patients without sputum as a symptom.

Two previous studies have evaluated the correlation between sputum quality and the culture results of *Legionella* species in *Legionella* pneumonia patients (27, 28). Ingram et al. investigated the correlation between sputum quality and culture results in 19 patients with *Legionella* pneumonia due to *L. pneumophila* (27). They reported that *L. pneumophila* was identified the most in Geckler six sputum (*n* = 7), followed by Geckler one sputum (*n* = 4), and most patients (78.9%) had non-purulent sputum (Geckler 1, 2, 3, and 6) (27). Another study by Shakeshaft et al. also showed that 46 of the 72 culture-positive *Legionella* pneumonia patients (63.9%) had non-purulent sputum, as defined by Murray and Washington criteria (28). The authors of these two studies concluded that all sputum samples, including non-purulent sputum, should be submitted for *Legionella* culture when *Legionella* pneumonia is suspected. The results of their studies were very significant for diagnosing *Legionella* pneumonia, although there were some possible limitations. First, only patients with identification of *Legionella* species by sputum culture were included in both studies. Therefore, the distribution of sputum quality and identification rate of *Legionella* species in each sputum quality group of *Legionella* pneumonia patients were unknown. Second, Ingram’s study included a relatively small number of patients (*n* = 19) (27), whereas most of the 72 culture-positive *Legionella* pneumonia patients included in Shakeshaft’s study were due to *L. longbeachae* (65.3%) (28). Therefore, our study is the first to evaluate the distribution of sputum quality and identification rate of *Legionella* species, including *L. pneumophila* and other species, according to sputum quality in over a hundred *Legionella* pneumonia patients, including more than 90% of *L. pneumophila* cases.

Regarding the correlation between pneumonia severity and the identification rate of *Legionella* species, our study showed that the identification rate was significantly higher in severe cases than in non-severe cases, as defined by PSI. There are several possible reasons for this observation. First, a previous report indicated that the sensitivity of *Legionella* UATs is higher in severe disease cases than in non-severe cases (29). According to that study, severe patients might have a greater load of *Legionella* species in sputum compared with urine. Second, it is possible that the attending physicians made a greater effort to collect sputum in severe pneumonia patients. However, in the present study, the collection rate of sputum was high (83.9%), and the collection rate of sputum was almost the same in PSI I-III and IV-V cases (75.0% vs 88.8%, data not shown). Therefore, sputum collection bias is not likely to have affected the results.

We also reported that administration of anti-*Legionella* antimicrobials before sputum evaluation was a negative predictor of the identification of *Legionella* species, and severe pneumonia, defined as PSI class ≥IV and ICU admission within 24 h after admission, was a positive predictive factor in the identification of *Legionella* species by sputum culture. A previous report showed that the sensitivity of sputum culture in diagnosing pneumococcal pneumonia decreased after antibiotic administration (93% without pre-culture antibiotics vs 74% with pre-culture antibiotics) (30). Another study by Mentasti et al. reported that the identification rate of *Legionella* species by sputum culture was higher in sputum collected within 2 days after admission compared with sputum collected more than 2 days after admission (79.6% vs 47.8%) (31). According to these reports, pre-culture antibiotic treatment with anti-*Legionella* antimicrobials might lead to a reduction in the amount of *Legionella* species observed in sputum. Indeed, our study showed that the identification rate of *Legionella* species significantly reduced from 24 h after administering anti-*Legionella* antibiotics. Musher et al. reported that the positive sputum culture rate for *S. pneumoniae* clearly decreased from 24 h after administering antibiotics compared with the rate within 24 h of antibiotic administration (28.6% in ≥24 h, 88.9% in 6–24 h, 77.8% in <6 h, and 93% without antibiotics) (30). In addition, despite a report of three cases of Legionnaires’ disease showing the correlation between positivity in polymerase chain reaction (PCR) tests and antibiotic treatment, Korosec et al. reported that *Legionella* amplicon intensity was highest in sputum or bronchial aspirates collected at or before the start of antibiotic therapy and decreased markedly within 3 days of antibiotic therapy (32). Therefore, sputum collection for culture should preferably be performed before or within 24 h of anti-*Legionella* antimicrobial administration, although treatment delay for sputum collection is not recommended. In terms of the correlation between identification rate and pneumonia severity, previous studies reported that the identification rate was higher in severe pneumonia compared with non-severe pneumonia cases, as previously mentioned (29). This is the first study to show that the identification rate was significantly higher in severe pneumonia, including cases requiring ICU admission within 24 h, than in non-severe pneumonia using multivariate analysis.

Regarding the diagnosis of *Legionella* pneumonia, gene tests, including PCR and LAMP, are essential for diagnosing *Legionella* pneumonia in patients with negative UAT results in daily clinical practice. Indeed, a previous report showed that a larger number of *Legionella* pneumonia patients could be diagnosed using both LAMP and UAT (33). However, PCR and LAMP could not detect the details of *Legionella pneumophila* SG and *Legionella* species. Therefore, performing sputum culture for *Legionella* species identification is likely to be significant for surveillance in individual areas and countries.

There are some limitations to the present study. First, this study was conducted retrospectively at a single center in Japan. Patients’ symptoms, including sputum, might have been underestimated due to its retrospective nature. Second, the attending physicians’ efforts in collecting sputum might have affected the diagnosis of *Legionella* pneumonia. *Legionella* pneumonia might have been underdiagnosed because the attending physicians did not collect sputum in pneumonia patients with negative *Legionella* UAT results because of the absence of sputum production as a symptom. Third, sputum samples were not obtained from 20 patients who were diagnosed with UATs. However, since the percentage of such patients was relatively small (16.1%), it is not likely to have affected the study results. Finally, since the prevalence of *Legionella* pneumonia and *Legionella* species varies between areas and countries, similar studies are needed in other countries as well. Additionally, since many *Legionella* pneumonia patients might be underdiagnosed due to negative UAT results, we believe that collection and culture of sputum for diagnosing *Legionella* pneumonia is important even in places with a low prevalence of *Legionella*. A strength of the present study is that it was relatively large, including 124 *Legionella* pneumonia patients, among whom sputum was collected from 104 patients. In addition, sputum quality was examined by experienced microbiological test engineers, and this is the first study to evaluate the identification rate of *Legionella* species in terms of sputum quality.

In conclusion, most *Legionella* pneumonia patients in this study had non-purulent sputum, and the identification rate of *Legionella* species was comparable between non-purulent and purulent sputum. These results suggest that regardless of sputum quality, sputum should be collected and cultured within 24 h of the administration of anti-*Legionella* antimicrobials in cases of suspected *Legionella* pneumonia.

## Data Availability

The data sets used and analyzed during the current study are available from the corresponding author upon reasonable request.
